# A symphony of immunity: How HPV mRNA-LNP vaccination orchestrates systemic anti-tumor responses

**DOI:** 10.1016/j.omtn.2025.102823

**Published:** 2026-01-12

**Authors:** Uri Elia, Dan Peer

**Affiliations:** 1 Department of Biochemistry and Molecular Genetics, Israel Institute for Biological Research, Ness-Ziona, Israel; 2 Laboratory of Precision Nanomedicine, Shmunis School of Biomedicine and Cancer Research, Tel Aviv University, Tel Aviv-Yafo, Israel; 3 Department of Materials Sciences and Engineering, Tel Aviv University, Tel Aviv-Yafo, Israel; 4 Center for Nanoscience and Nanotechnology, Tel Aviv University, Tel Aviv-Yafo, Israel; 5 Cancer Biology Research Center, Tel Aviv University, Tel Aviv-Yafo, Israel

The remarkable success of mRNA vaccines in recent years has put the mRNA lipid nanoparticle (LNP) platform in the forefront of developing highly effective countermeasures against infectious diseases. Nevertheless, mRNA-LNPs have been studied for over three decades in the context of cancer therapy,^1^ and mRNA cancer vaccines are now being extensively pursued owing to recent advancements in this field.^2^^,^^3^ Although an anti-tumor effect mediated by mRNA-LNPs necessitates coordinated, multi-compartment, humoral and cellular immune responses, these crucial immune events have not been fully characterized. In a recent study published in Molecular Therapy Nucleic Acids, Qiu et al.^4^ provides a detailed look into the systemic anti-tumor immune responses orchestrated by mRNA-LNPs. Using a murine model of human papillomavirus (HPV)-positive head and neck squamous cell carcinoma (HNSCC), the authors highlight the spatiotemporal cellular and molecular processes that underlie the anti-tumor effects of mRNA-LNP vaccination and provide valuable insights into potential cellular targeting for optimization of vaccine efficacy. Their study goes beyond the classical focus on T cell priming and effector differentiation to describe how B cells, myeloid cells, and innate-like lymphocytes collectively integrate innate interferon (IFN) signals and antigen-driven proliferation into a coordinated anti-tumor response.

mRNA vaccines hold several advantages over conventional vaccine platforms (e.g., subunit, live attenuated, etc.), making them extremely versatile and relevant for cancer immunotherapy; mRNA vaccines can be rapidly produced in a cell-free manner, and the encoding antigen of choice can be easily designed and replaced.^5^ In addition, mRNA-LNPs have been shown to activate both innate and adaptive immune responses and to elicit both humoral and cellular immunity, enabling not only direct, local tumor effects but also remote lymphatic organ modulation. Taken together, these features make mRNA-LNP a highly suitable candidate for combating different malignancies, as evidenced by the large number of cancer mRNA vaccine clinical trials.^6^ Despite the accumulating knowledge regarding the different immune responses, cell populations, and signaling pathways affected by mRNA vaccines ultimately leading to anti-tumor activity, a detailed map of these concerted spatiotemporal processes has yet to be appropriately characterized.

The authors previously demonstrated the anti-tumor efficacy of HPV mRNA-LNPs, reporting that a burst of CD8+ T cells mediated tumor control and regression.^7^ Now, Qiu and colleagues sought to deepen the understanding of the systemic immune events that occur at different stages and different locations following vaccination, ultimately facilitating these anti-tumor effects. To characterize immune cell composition shifts following vaccination, immune cells from tumors, blood, tumor-draining lymph nodes (TDLNs), and spleens were evaluated, enabling the identification of several subsets of CD8+ T cells, CD4+ T cells, B cells, and myeloid cells. By incorporating single-cell RNA sequencing to the analysis, the authors were able to portray a multifaceted interaction between the immune cell subtype, its anatomical location, and transcriptional signature. Upon vaccination, spleens were characterized by a decrease in activated T cells and B cells and accumulation of antigen-presenting cells (APCs), while blood analysis showed rapid increase in activated B cells, likely migrating from the spleen via the circulation. Additionally, TDLNs were characterized by a rapid increase in IFN stimulated genes (ISG)-expressing T cells, while tumor microenvironment (TME) displayed enrichment of effector memory and exhausted CD8+ T cells. These sequential coordinated immune events support the development of effective anti-tumor immunity.

ISG signature of immune cells represents a rapid, immune-alert state induced by type I IFNIFN-I signaling, transforming immune cells into highly responsive effectors that amplify innate detection, antigen presentation, and adaptive priming.^8^ In an unexpected and striking discovery, Qiu et al. found that ISG signature was induced not only in CD4+ and CD8+ T cells but also in γδ T cells and multiple B cell subsets. Interestingly, proportions of all these subsets rise sharply in TDLNs, suggesting that these nodes function as an early “IFN amplifier” after systemic mRNA-LNP vaccination. Importantly, the authors show that this ISG signature was recapitulated using an empty LNP vector, suggesting that the observed IGS signature following vaccination originates from non-antigen-specific properties of LNP components (Figure 1). Indeed, such adjuvant effect is in line with recent findings,^9^ demonstrating LNP-mediated activation of TLRs. While this non-antigen-specific ISG activation can facilitate T cell priming, enhancing subsequent antigen-specific immune responses, excessive IFN-I signaling could lead to T cell exhaustion and an overall reverse effect. Therefore, fine-tuning LNP components in order to adjust the magnitude of these effects should be taken into considerations when optimizing cancer mRNA vaccine design.

**Figure 1:**
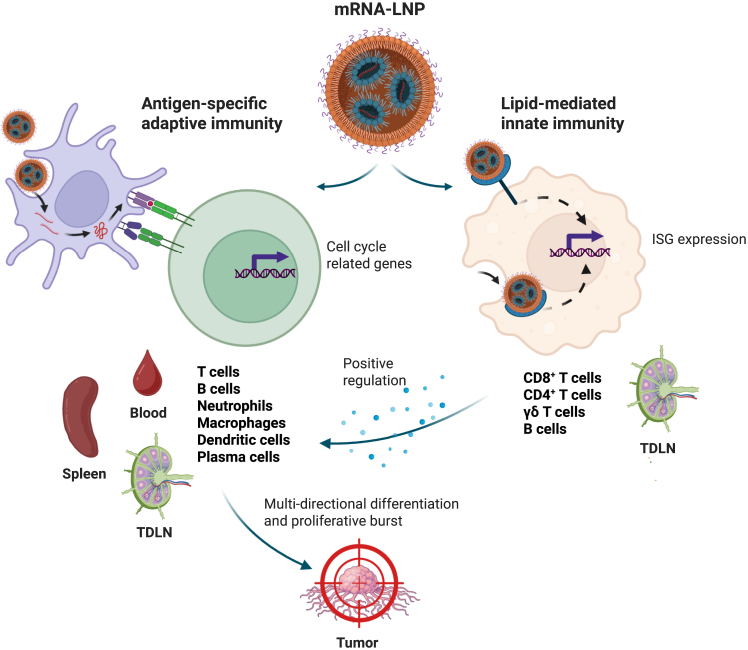
mRNA-LNP vaccine orchestrates systemic immunity by coupling lipid-mediated innate interferon signaling with systemic, antigen-specific-driven proliferation, and multi-directional differentiation of immune cells Image created with BioRender.

T cell and B cell activation and proliferation are considered hallmarks of successful vaccine-induced immune response^10^ and, as expected, were identified in the current study following HPV mRNA-LNP vaccination. Intriguingly, Qiu et al. discovered that myeloid cells constitute a large proportion of the cycling compartment as well, implying that mRNA-LNP immunization led to an unexpected proliferative surge of innate immune system components. Furthermore, these cells were found to be capable of multi-directional differentiation, enabling activation of antigen processing pathways within secondary lymphoid organs or tumor necrosis factor (TNF), nuclear factor κB (NF-kB), and chemokine signaling pathways within the TME. Notably, this observation marks myeloid cells as key contributors to the anti-tumor immunity of mRNA-LNP vaccines, bridging between early innate IFN-driven response and the later antigen-specific adaptive response (Figure 1). An additional interesting finding was the critical role of B cells in anti-tumor response. The data demonstrated the rapid migration of B cells to secondary lymphoid organs and formation of germinal center (GC) cell populations, facilitating antigen presentation and supporting CD4+ and CD8+ T cell priming. In the TME, B cells upregulated pathways related to FcγR signaling and B cell receptor activation, indicating major roles in both humoral and cellular immunity.

Several limitations and open questions in this study should be considered, some of them raised by the authors. First, to minimize estrous variability and reflect male-biased incidence of HNSCC, the study employed male C57BL/6 mice only, neglecting the possibility of sex differences in mRNA vaccine responsiveness.^11^ Moreover, the complex immune responses and modulations in immune cell populations recorded in this study may be affected by the genetic background of the model, for example C57BL/6 vs. BALB/c mice.^12^ In addition, the mRNA-LNP vaccine was administered intravenously and may present different pharmacokinetics, biodistribution, and expression profile when compared to other clinical administration routes.^13^ Lastly, the LNP formulation used in this study is based on the MC3 ionizable lipid. Although stated by the authors as sharing critical design principles with clinically approved ionizable lipids, a direct head-to-head comparison would be required for generalization of the data to other LNP chemistries.

Looking forward, the study by Qiu et al. sets the stage for future exploration. First, dissecting the functional role of ISG-positive subsets via conditional IFN signaling knockouts, or via pharmacologic modulation of IFN pathways during vaccination, would clarify whether these cells simply correlate with, or are essential for, durable tumor control. Second, rational LNP engineering can be used to control innate activation kinetics, tailoring ISG induction to desired therapeutic windows and thereby increasing vaccine efficacy. Moreover, extending the analysis to additional tumor models and to alternative LNP chemistries and administration routes would determine how generalizable the systemic coordination principles are across cancers and platforms.

In summary, Qiu et al. provide a comprehensive demonstration that mRNA-LNP vaccines function not only as antigen delivery vectors but also as orchestrators of systemic immune remodeling. Their work paves the way for finely tuned immunotherapies capable of reshaping entire immune landscapes, ultimately optimizing vaccine efficacy.

## Declaration of interests

D.P. receives licensing fees (to patents on which he was an inventor) from, invested in, consults (or on scientific advisory boards or boards of directors) for, lectured (and received a fee) or conducts sponsored research at Tel Aviv University for the following entities: ART Biosciences, BioNtech SE, Earli Inc., Kernal Biologics, LAND Therapeutics, Merck KGaA, Newphase Ltd., NeoVac Ltd., RiboX Therapeutics, Roche, SirTLabs Corporation, and Teva Pharmaceuticals Inc.
